# Everyday and everynight psychiatry - experiencing a ward cover shift through zoom

**DOI:** 10.1192/j.eurpsy.2021.1589

**Published:** 2021-08-13

**Authors:** A. Fukutomi, M. Bhamra, S. Butler, C. Wilson-Jones

**Affiliations:** Undergraduate Psychiatry Training, South London and Maudsley NHS Foundation Trust, London, United Kingdom

**Keywords:** Zoom, online, Teaching, undergraduate

## Abstract

**Introduction:**

The delivery of medical education has changed alongside the effects of COVID-19. As a result, the undergraduate psychiatry training for medical students at Guy’s King’s and St Thomas’ School of Medicine had to adapt rapidly. This poster portrays the journey in which the teaching sessions were developed and delivered throughout the first academic term of 2020-2021.

**Objectives:**

To deliver an interactive online teaching day that can provide students with the knowledge and understanding of common psychiatric disorders in the interface of other medical conditions.

**Methods:**

A clinical skills teaching day was developed to deliver the sessions via the online video calling platform Zoom. Published articles regarding online medical education as well as guidelines from the Royal College of Psychiatry were used as a resource to develop the structure. Feedback of the teaching day was collected via an anonymous survey.

**Results:**

78 responses were collected in total from 4 teaching days. Overall satisfaction was high with a score of 86.5/100 in overall satisfaction. Themes for positive feedback included utilising actors in simulation (38% 30/78) and high interactivity within the teaching (31% 24/78). There were a number of students who found the whole day session online tiring (13% 10/78) and others felt the variation of scenarios were too limited (12% 9/78).

**Conclusions:**

As lockdown has forced students to have less patient contact, they have suffered from the lack of learning opportunities. This teaching day showed the importance of organising high fidelity scenarios in order to try and fill the void that has been created due to COVID-19.

